# Supplementary material for: Emergent patterns of reef fish diversity correlate with coral assemblage shifts along the Great Barrier Reef
Source: Nat Commun. 2025 Jan 13;16:303. doi: 10.1038/s41467-024-55128-7 (PMC11729903; doi:10.1038/s41467-024-55128-7)
Supplement: Supplementary file 1 — Supplementary Information [file 41467_2024_55128_MOESM1_ESM.pdf]

Supporting Information of Gonzalez-Barrios et al. *Emergent patterns of reef fish diversity correlate with coral assemblage shifts along the Great Barrier Reef*

Supplementary Table 1. **Sample size of reefs for every latitudinal sector and year.** Surveys were structured hierarchically. Within each reef, three sites were surveyed and five transects were used in each site. Codes for latitudinal sectors are Cooktown-Lizard Island (CL), Cairns (CA), Innisfail (IN), Townsville (TO), Whitsunday (WH), Pompey (PO), Swain (SW) and Capricorn-Bunker (CB).

	CA	*CB*	*CL*	*IN*	*PO*	*SW*	*TO*	*WH*
1995	10	3	8			7	7	9
1996	10	4	8			7	7	9
1997	9	4	8			7	8	8
1998	10	4	8			7	8	9
1999	10	4	8			7	8	9
2000	10	4	8			7	8	9
2001	9	4	8			7	8	9
2002	10	4	8			7	8	9
2003	10	4	8			7	8	9
2004	10	4	8			7	8	9
2005	10	4	8			7	8	9
2006	5	8		7	12	12	12	
2007	10	4	8			6	8	9
2008	5	8		7	12	12	12	
2009	10	4	8			7	8	9
2010	5	8		7	12	12	12	
2011	10	4	8			7	8	9
2012	5	8		7	12	12	12	
2013	10	4	8			7	8	9
2014	5	8		3	12	12	10	
2015	10	4	8			7	8	9
2016	5	8		7	12	12	12	
2017	11	4	8			7	8	9
2018	5	8		7	12	10	12	
2019	10	4	8			7	7	8
2020	5	8		7	10	8	14	
2021	10	4	8			7	8	9
2022	6	7	8	4	8	9	10	7

Supplementary Table 2. **Coral groups/ taxa used from the Australian Institute of Marine Science' Long Term Monitoring Program.**

Coral groups
Acanthastea, Micromussa, Homophyllia
Acropora branching & bottlebrush
Acropora digitate

Acropora submassive & encrusting	
Acropora tabulate & corymbose	
Agariciidae encrusting & submassive	
Agariciidae foliose	
Astreopora	
Diploastrea	
Echinopora branching	
Echinopora other	
Fungiidae encrusting & foliose	
Fungiidae free-living	
Galaxea	
Goniopora, Alveopora & Bernardpora	
Hydnophora branching	
Hydnophora not branching	
Isopora branching	
Isopora encrusting-submassive	
Leptastrea	
Lobophyllia, Australophyllia & A. pachysepta	
Merulina	
Merulinidae encrusting, massive & sub-massive	
Merulinidae, Plesiastreidae plocoid	
Montipora	
Moseleya	
Mycedium	
Oxypora, Echinophyllia, Echinomorpha	
Pachyseris	
Pectinia	
Plerogyridae and Euphylliidae	
Pocillopora	
Porites branching	
Porites encrusting & sub-massive	
Porites massive	
Porites rus	
Seriatopora	
Siderastreidae, Coscinaraeidae, Psammocoridae	
Stylophora	
Tubastrea, Heteropsammia	
Turbinaria and Duncanopsammia	

Supplementary Table 3. **Annual average of species richness (SR) ± Standard deviation (SD) for every latitudinal sector and year.** Codes for latitudinal sectors are Cooktown-Lizard Island (CL), Cairns (CA), Innisfail (IN), Townsville (TO), Whitsunday (WH), Pompey (PO), Swain (SW) and Capricorn-Bunker (CB).

Year		CA	CB	CL	IN	PO	SW	TO	WH
1995	SR	45.7	22.3	53.8			42.6	47.6	37.1
	SD	7.0	13.1	6.3			5.7	12.2	8.6
1996	SR	46.9	26.0	56.4			44.2	46.7	43.1
	SD	6.7	9.9	6.5			7.4	11.4	9.0
1997	SR	40.2	30.7	56.3			42.6	44.3	37.5
	SD	7.0	12.4	5.6			5.4	13.4	14.4
1998	SR	45.1	37.1	56.3			42.4	42.6	38.1
	SD	7.0	9.6	6.5			4.6	11.0	12.7
1999	SR	45.1	40.6	55.3			42.9	41.0	43.7
	SD	5.9	8.1	6.1			7.0	15.6	10.7
2000	SR	46.7	44.8	56.3			46.0	41.0	39.9
	SD	7.0	5.6	6.5			5.6	12.8	9.0
2001	SR	44.3	40.8	57.1			43.0	41.6	39.9
	SD	6.5	5.5	6.6			5.8	11.7	9.7
2002	SR	44.6	45.1	57.1			42.8	42.4	39.1
	SD	6.9	6.4	6.9			7.4	13.3	9.7
2003	SR	47.3	49.6	57.7			42.6	44.3	43.0
	SD	9.3	3.0	5.6			5.8	12.7	10.2
2004	SR	46.7	47.1	59.2			44.0	43.8	43.4
	SD	8.1	4.7	5.8			5.1	13.4	8.4
2005	SR	46.4	51.5	60.3			48.0	44.5	41.9
	SD	6.0	5.1	6.8			7.3	13.2	11.2
2006	SR	46.7	50.9		47.8	39.5	48.3	50.2	
	SD	6.3	7.5		6.9	6.0	6.6	7.1	
2007	SR	46.3	50.8	60.4			44.5	43.2	43.1
	SD	7.7	7.2	6.3			3.9	14.8	10.4
2008	SR	49.2	47.9		51.7	38.0	47.7	47.9	
	SD	4.9	6.3		6.7	5.9	7.5	6.4	
2009	SR	46.5	42.3	60.6			44.8	45.5	44.3
	SD	7.3	10.1	5.8			6.2	14.1	12.2
2010	SR	49.9	42.2		50.1	41.1	48.6	48.9	
	SD	3.8	11.9		4.9	6.3	9.3	6.9	
2011	SR	45.7	25.5	58.8			44.0	42.7	40.3
	SD	8.4	12.9	7.9			7.8	13.2	10.3
2012	SR	51.7	38.0		50.5	40.5	48.6	48.9	
	SD	5.5	12.6		7.4	5.5	6.7	5.7	
2013	SR	46.1	24.4	60.2			40.0	42.6	43.8
	SD	8.5	10.4	8.8			6.6	16.6	9.1
2014	SR	49.1	37.7		50.4	37.4	46.3	48.0	
	SD	5.7	11.2		7.6	6.5	7.7	7.3	
2015	SR	44.2	25.3	54.3			40.0	41.8	41.5
	SD	7.8	7.9	8.4			6.2	15.0	9.1
2016	SR	45.8	42.3		51.9	37.7	48.4	50.7	
	SD	4.0	9.6		5.3	6.6	8.0	6.8	
2017	SR	42.2	36.7	51.1			43.2	41.0	41.0
	SD	7.1	6.5	8.7			6.6	13.5	9.5
2018	SR	45.9	40.2		51.8	36.0	45.8	46.4	

	SD	3.5	10.5		4.3	5.8	6.4	6.6	
2019	SR	40.9	44.2	49.2			41.7	39.3	38.2
	SD	9.9	5.1	8.8			6.4	15.0	11.2
2020	SR	45.4	44.0		47.1	35.8	48.9	46.8	
	SD	4.7	8.5		7.6	4.5	5.4	5.9	
2021	SR	40.8	39.8	52.4			44.5	43.0	40.6
	SD	9.0	5.9	7.0			5.1	14.6	10.6
2022	SR	36.8	45.9	52.2	44.7	39.0	42.0	43.2	44.4
	SD	5.9	5.2	6.0	5.4	4.9	4.4	11.9	9.2

Supplementary Table 4. **Model structures.** All models were fitted to normal distributions *N* (μ_i, σ) with *'reef'* and *'shelf-position'* as random intercepts (u_{0i}). In models 1 to 5, 8 and 9 we added a correlation structure of the standard class autoregressive process of order 1 (corAR1) to control for possible temporal autocorrelation, adding '*year'* as the correlation variable.

Model	Type	Response	Structure
1	HGAM	Species richness	$(\beta 0 + u_{0i}) + \beta_1\,(latitude)_i \times \beta_2\,(periods)_i + \varepsilon_i$
2	HGAM	β year-to-year	$(\beta 0 + u_0) + \beta_1\,(year)_i + \varepsilon_i$
3	HGAM	β year-to-year	$(\beta 0 + u_{0i}) + \beta_1\,(year)_i \times \beta_2\,(sectors)_i + \varepsilon_i$
4	HGAM	β reference	$c(\beta 0 + u_0) + \beta_1\,(year)_i + \varepsilon_i$
5	HGAM	β reference	$(\beta 0 + u_{0i}) + \beta_1\,(year)_i \times \beta_2\,(sectors)_i + \varepsilon_i$
6	GLMM	β year-to-year	$(\beta 0 + u_{0i}) + \beta_1\,(periods)_i + \varepsilon_i$
7	GLMM	β reference	$(\beta 0 + u_{0i}) + \beta_1\,(periods)_i + \varepsilon_i$
8	HGAM	Coral cover	$(\beta 0 + u_{0i}) + \beta_1\,(year)_i + \varepsilon_i$
9	HGAM	Coral composition	$(\beta 0 + u_{0i}) + \beta_1\,(year)_i + \varepsilon_i$
10	GLMM	Coral cover	$(\beta 0 + u_{0i}) + \beta_1\,(periods)_i + \varepsilon_i$
11	GLMM	Coral composition	$(\beta 0 + u_{0i}) + \beta_1\,(periods)_i + \varepsilon_i$
12	GLMM	β year-to-year	$(\beta 0 + u_{0i}) + \beta_1\,(\Delta\, coral\ cover)_i + \beta_2\,(\Delta\, coral\ composition)_i + \varepsilon_i$
13	GLMM	Δ Species richness	$(\beta 0 + u_{0i}) + \beta_1\,(\Delta\, coral\ cover)_i + \beta_2\,(\Delta\, coral\ composition)_i + \varepsilon_i$
14	GLMM	β year-to-year	$(\beta 0 + u_{0i}) + \times \beta_1\,(\Delta\, coral\ composition)_i \times \beta_2\,(\Delta\, sectors)_i + \varepsilon_i$
15	GLMM	β year-to-year	$(\beta 0 + u_{0i}) + \times \beta_1\,(\Delta\, coral\ cover)_i \times \beta_2\,(\Delta\, sectors)_i + \varepsilon_i$
16	GLMM	Δ Species richness	$(\beta 0 + u_{0i}) + \times \beta_1\,(\Delta\, coral\ composition)_i \times \beta_2\,(\Delta\, sectors)_i + \varepsilon_i$

Supplementary Table 5. **Fish species surveyed by functional group from the Australian Institute of Marine Science' Long Term Monitoring Program.**

Group	N	Species
Carnivore	27	*Aethaloperca rogaa, Anyperodon leucogrammicus, Lethrinus atkinsoni, Lethrinus erythracanthus, Lethrinus miniatus, Lethrinus nebulosus, Lethrinus obsoletus, Lethrinus olivaceus, Lethrinus rubrioperculatus, Lethrinus xanthochilus, Lutjanus adetii, Lutjanus argentimaculatus, Lutjanus biguttatus, Lutjanus bohar, Lutjanus carponotatus, Lutjanus fulvus, Lutjanus gibbus, Lutjanus kasmira, Lutjanus lemniscatus, Lutjanus lutjanus, Lutjanus monostigma, Lutjanus quinquelineatus, Lutjanus rivulatus, Lutjanus russellii, Lutjanus sebae, Lutjanus semicinctus, Lutjanus vitta*
Corallivore	27	*Bolbometopon muricatum, Chaetodon aureofasciatus, Chaetodon auriga, Chaetodon baronessa, Chaetodon bennetti, Chaetodon citrinellus, Chaetodon ephippium, Chaetodon flavirostris, Chaetodon kleinii, Chaetodon lineolatus, Chaetodon lunul, Chaetodon melannotus, Chaetodon meyeri, Chaetodon ornatissimus, Chaetodon pelewensis, Chaetodon plebeius , Chaetodon punctatofasciatus, Chaetodon rafflesii, Chaetodon rainfordi, Chaetodon reticulatus, Chaetodon speculum, Chaetodon trifascialis, Chaetodon trifasciatus, Chaetodon ulietensis, Chaetodon unimaculatus, Chaetodon vagabundus, Plectroglyphidodon johnstonianus*
Detritivore	4	*Acanthurus dussumieri, Acanthurus olivaceus, Acanthurus xanthopterus, Ctenochaetus spp*

Herbivore	57	*Acanthurus maculiceps, Acanthurus nigricans, Acanthurus nigricauda, Acanthurus nigrofuscus, Acanthurus pyroferus, Acanthurus triostegus, Calotomus carolinus, Cetoscarus ocellatus, Chlorurus bleekeri, Chlorurus japanensis, Chlorurus microrhinos, Chlorurus sordidus, Chrysiptera biocellata, Dischistodus melanotus, Dischistodus perspicillatus, Dischistodus prosopotaenia, Dischistodus pseudochrysopoecilus, Hipposcarus longiceps, Naso lituratus, Naso tonganus, Naso unicornis, Plectroglyphidodon lacrymatus, Pomacentrus adelus, Pomacentrus chrysurus, Pomacentrus grammorhynchus, Pomacentrus tripunctatus, Pomacentrus wardi, Scarus altipinnis, Scarus chameleon, Scarus dimidiatus, Scarus flavipectoralis, Scarus forsteni, Scarus frenatus, Scarus ghobban, Scarus globiceps, Scarus niger, Scarus oviceps, Scarus psittacus, Scarus rivulatus, Scarus rubroviolaceus, Scarus schlegeli, Scarus spinus, Siganus argenteus, Siganus corallinus, Siganus doliatus, Siganus lineatus, Siganus puellus, Siganus punctatissimus, Siganus punctatus, Siganus spinus, Siganus vulpinus, Stegastes apicalis, Stegastes fasciolatus, Stegastes gascoynei, Stegastes nigricans, Zebrasoma scopas, Zebrasoma veliferum*
Invertivore	14	*Cheilinus fasciatus, Cheilinus undulatus, Choerodon fasciatus, Coris gaimard, Epibulus insidiator, Forcipiger flavissimus, Forcipiger longirostris, Gomphosus varius, Halichoeres hortulanus, Hemigymnus fasciatus, Hemigymnus melapterus, Lethrinus harak, Monotaxis grandoculis, Zanclus cornutus*
Omnivore	24	*Acanthochromis polyacanthus, Amblyglyphidodon curacao, Amblyglyphidodon leucogaster, Amphiprion akindynos, Amphiprion chrysopterus, Amphiprion clarkii, Amphiprion melanopus, Amphiprion percula, Amphiprion perideraion, Chaetodon mertensii, Chelmon rostratus, Chrysiptera rex, Neoglyphidodon melas, Neoglyphidodon nigroris, Plectroglyphidodon dickii, Pomacentrus amboinensis, Pomacentrus australis, Pomacentrus bankanensis, Pomacentrus brachialis, Pomacentrus moluccensis, Pomacentrus nagasakiensis, Pomacentrus pavo, Pomacentrus vaiuli, Premnas biaculeatus*
Piscivore	7	*Cephalopholis sexmaculata, Plectropomus areolatus, Plectropomus laevis, Plectropomus leopardus, Plectropomus maculatus, Variola albimarginata, Variola louti*
Planktivore	38	*Acanthurus albipectoralis, Acanthurus mata, Acanthurus thompsoni, Amblyglyphidodon aureus, Chromis acares, Chromis agilis, Chromis amboinensis, Chromis atripectoralis, Chromis atripes, Chromis chrysura, Chromis iomelas, Chromis lepidolepis, Chromis lineata, Chromis margaritifer, Chromis nitida, Chromis retrofasciata, Chromis ternatensis, Chromis vanderbilti, Chromis viridis, Chromis weberi, Chrysiptera flavipinnis, Chrysiptera rollandi, Chrysiptera talboti, Dascyllus aruanus, Dascyllus melanurus, Dascyllus reticulatus, Dascyllus trimaculatus, Hemitaurichthys polylepisl, Neoglyphidodon polyacanthus, Neopomacentrus azysron, Neopomacentrus bankieri, Neopomacentrus cyanomos, Paracanthurus hepatus, Pomacentrus coelestis, Pomacentrus lepidogenys, Pomacentrus philippinus, Pomacentrus reidi, Pomachromis Richardson*

Supplementary Table 6. **Net contribution to community dissimilarity (*net* =) for each functional group.** Net contribution is the balance between the negative and positive contributions to community dissimilarity between the initial period of each period and the more recent (2016-2022) from the SIMPER analysis. Codes for latitudinal sectors are Cooktown-Lizard Island (CL), Cairns (CA), Innisfail (IN), Townsville (TO), Whitsunday (WH), Pompey (PO), Swain (SW) and Capricorn-Bunker (CB).

Functional group	CL	CA	IN	TO	WH	PO	SW	CB
Omnivore	-4.46	-2.19	-1.19	-1.07	-4.01	-4.55	-0.121	1.83
Planktivore	-4.9	-2.88	-2.19	-0.00974	-0.501	-2.62	-2.98	0.346
Corallivore	-2.64	-0.754	0.521	-1.54	-0.0876	-1.51	0.382	-0.993
Invertivore	-1.05	-0.66	-0.648	-0.244	-0.285	-0.864	-0.279	0.29
Herbivore	-1.54	-3.05	-1.6	2.51	0.191	-3.58	0.685	6.95
Carnivore	0.365	-1.38	0.572	0.0696	0.101	0.274	-0.0143	1.1
Detritivore	0.117	-0.269	-0.138	0.115	0.422	0.219	0.141	0.598
Piscivore	0.326	0.107	0.0234	0.134	0.303	0.131	-0.0243	0.353

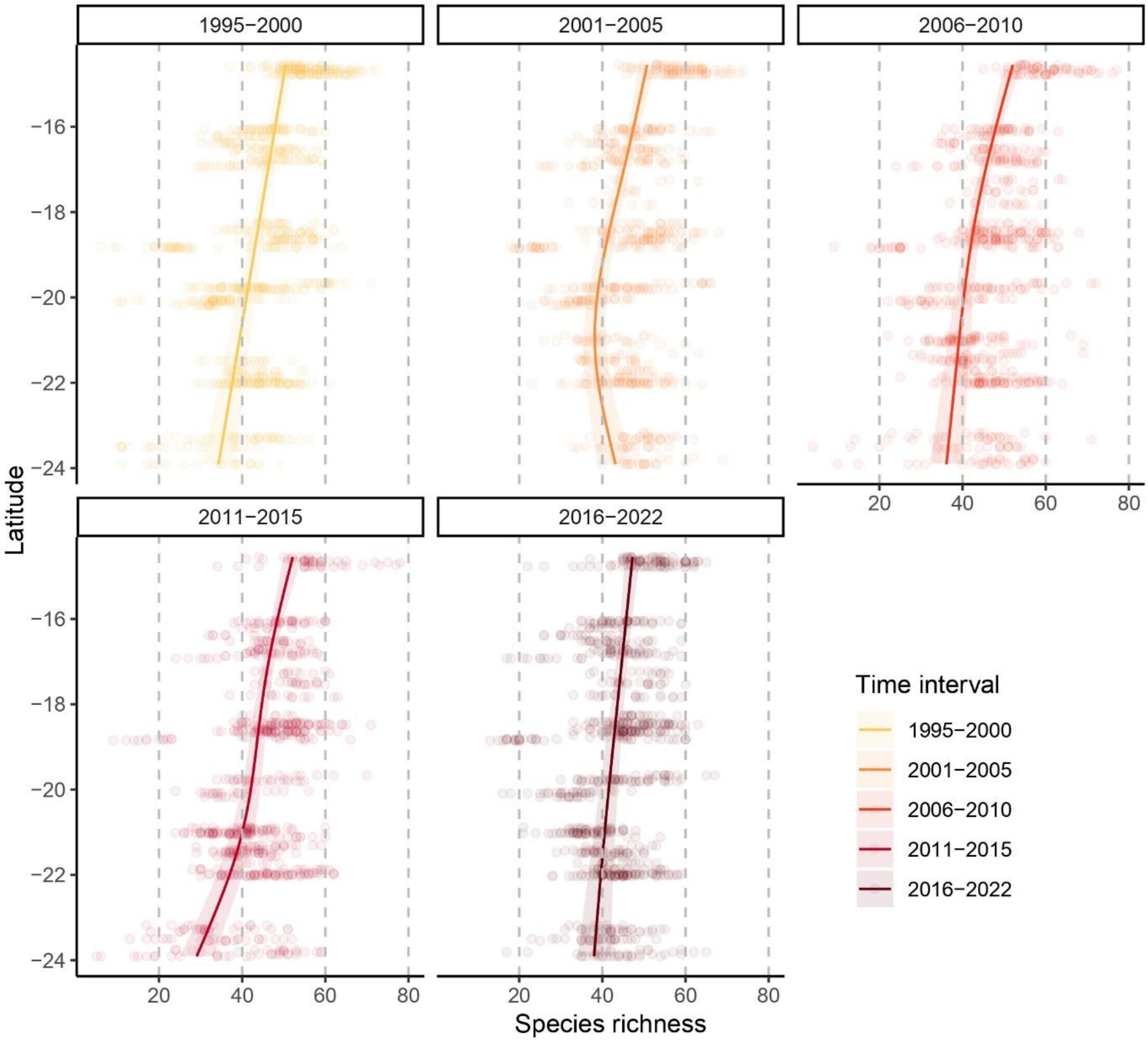

Supplementary Figure 1. **Species richness (α diversity) of reef fish across time and latitude on the GBR.** Species richness patterns of 92 reefs (three sites per reef per year) across the latitudinal gradient based on predicted values of species richness in five-time intervals (1995-2000, 2001-2005, 2006-2010, 2011-2015 and 2016-2022) from the hierarchical generalized additive mixed model (HGAM). Dots represent the species richness of each site and lines represent the fitted model (shading shows the 95% CIs). Gradient colour from yellow to red, representing the initial period to the more recent one. Source data are provided as a Source Data file.

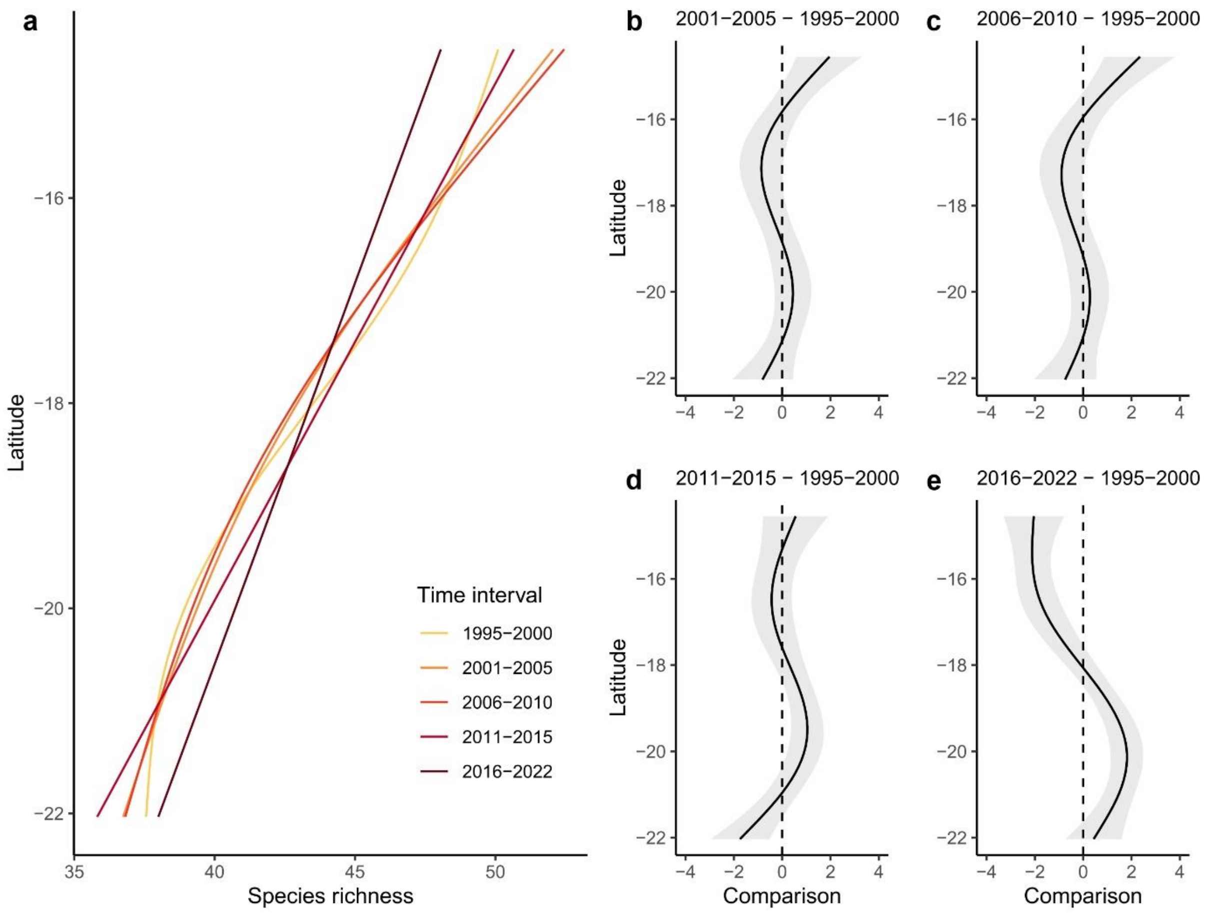

Supplementary Figure 2. **Species richness (α diversity) of reef fish across time and latitude on the GBR without the Capricorn-Bunker sector (n = 82 reefs with three sites each per year).** Species richness pattern across the latitudinal gradient (**a**) based on predicted values of species richness in five-time intervals (1995-2000, 2001-2005, 2006-2010, 2011-2015 and 2016-2022) from the hierarchical additive mixed model (HGAM). Each model line is shown in a gradient colour from yellow to red, representing the initial period to the more recent one, respectively. Grey bands in (**b**)-(**e**) represent the 95% CIs. Comparison of the number of fish species with the initial time period (i.e., 1995-2000) among all other subsequent periods are shown in (**b**) 2001-2005, (**c**) 2006-2010, (**d**) 2011-2015 and (**e**) 2016-2022. Source data are provided as a Source Data file.

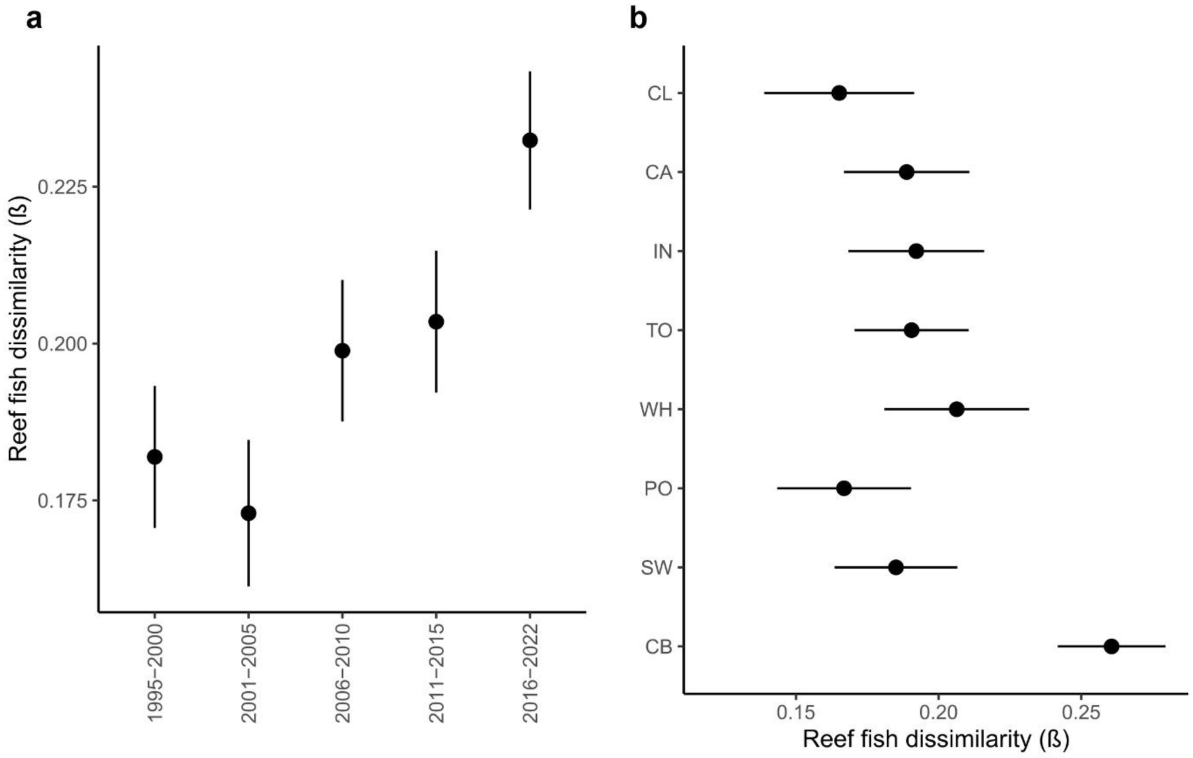

Supplementary Figure 3. **Sensitive analysis of reef fish turnover (*β* diversity) of reefs that were biennially surveyed (n = 82 with three sites each per year) across the GBR and the latitudinal sectors.** Predicted values from the generalized linear mixed model (GLMM) are shown in (**a**) for the GBR model and values of the hierarchical generalized additive mixed model (HGAM) are shown in (**b**) for the latitudinal sector. Number of reefs per time period in (**a**): 1995-2000 (*n* reefs = 36), 2001-2005 (*n* reefs = 36), 2006-2010 (*n* reefs = 82), 2011-2015 (*n* reefs = 82), and 2016-2022 (*n* reefs = 82). Points represent the mean predicted values and lines represent the 95% CIs. Latitudinal sectors are arranged in descending order from low to high latitudes. Codes for latitudinal sectors are Cooktown-Lizard Island (CL), Cairns (CA), Innisfail (IN), Townsville (TO), Whitsunday (WH), Pompey (PO), Swain (SW) and Capricorn-Bunker (CB). Source data are provided as a Source Data file.

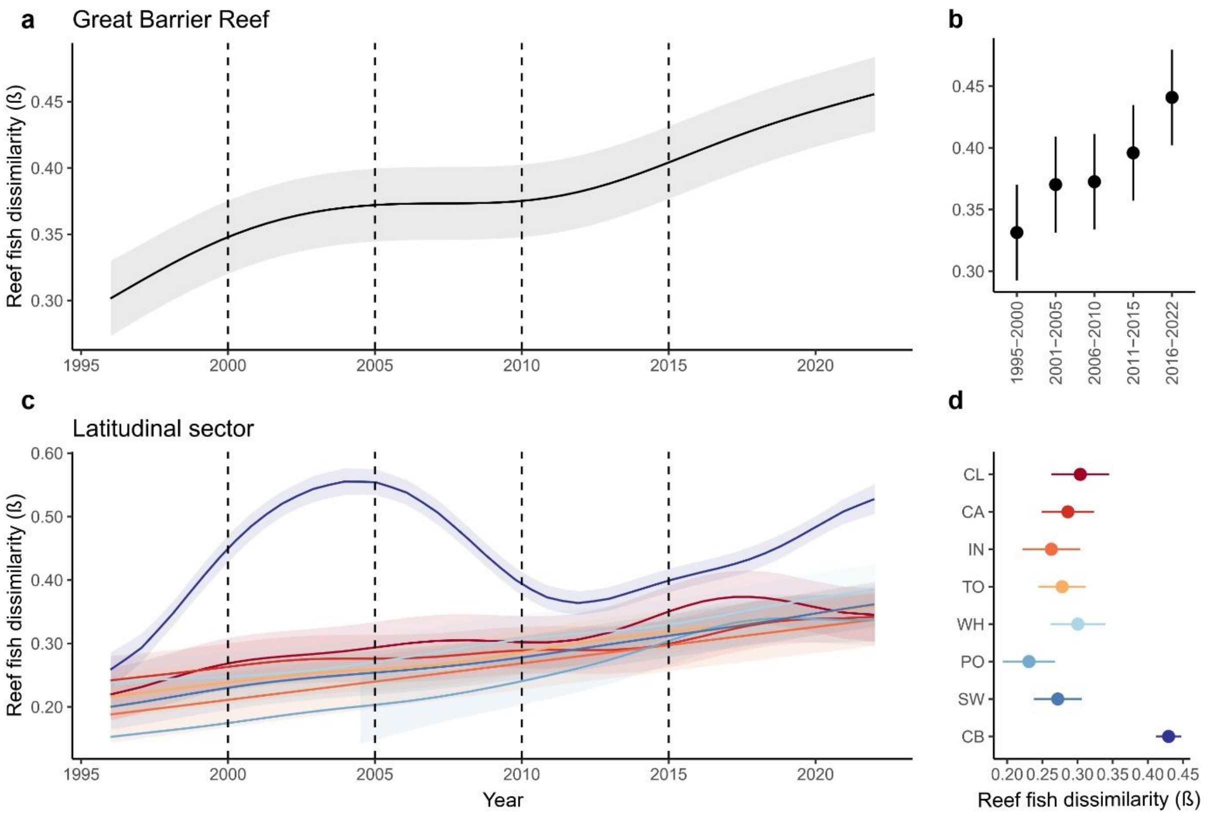

Supplementary Figure 4. **Reef fish reference-year dissimilarity (*β* diversity) across the Great Barrier Reef (GBR) and the latitudinal sectors.** Predicted values of the long-term changes in the reef fish dissimilarity of 92 reefs (three sites per reef per year) from hierarchical generalized additive mixed models (HGAM) according to the initial sampling year in the GBR (**a**) and across the latitudinal sectors (**c**). Shadow bands in (**a**) and (**c**) represent the 95% CIs. Latitudinal sectors in (**d**) are arranged from low to high latitudes and in colours from red to blue, respectively. Predicted values from the generalized linear mixed model (GLMM) are shown in (**b**) for the GBR model and from the HGAM are shown in (**d**) for the latitudinal sectors. Points represent the mean of the predicted values and lines represent the 95% CIs. Latitudinal sectors are arranged in descending order from north to south and in colours from red to blue, respectively. Codes and number of reefs for latitudinal sectors are Cooktown-Lizard Island (CL, *n* reefs = 8), Cairns (CA, *n* reefs = 11), Innisfail (IN, *n* reefs = 7), Townsville (TO, *n* reefs = 18), Whitsunday (WH, *n* reefs = 9), Pompey (PO, *n* reefs = 12), Swain (SW, *n* reefs = 17) and Capricorn-Bunker (CB, *n* reefs = 10). Source data are provided as a Source Data file.

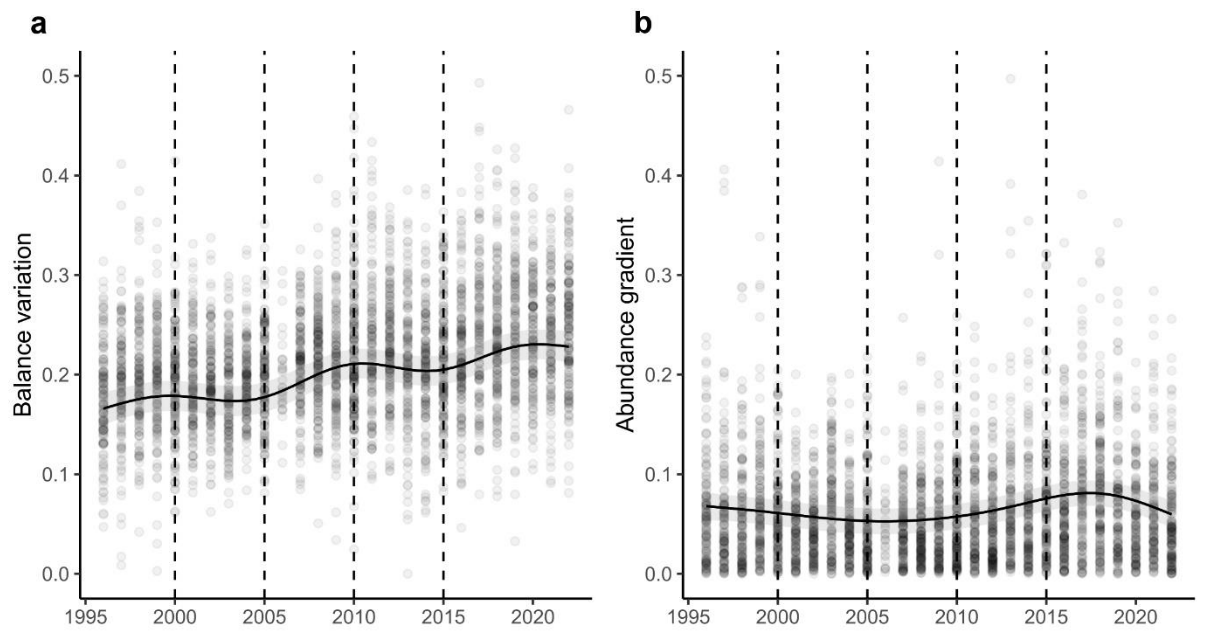

Supplementary Figure 5. **Reef fish year-to-year dissimilarity (*β* diversity) in the Great Barrier Reef.** Trends are the predicted values ± 95% CIs of the long-term changes of 92 reefs (three sites per reef per year) in the reef fish dissimilarity from the hierarchical generalized additive mixed model (HGAM) are separated into two components accounting for the dissimilarity derived solely from (**a**) balance variation (i.e., turnover), and (**b**) the dissimilarity derived from abundance gradient (i.e., nestedness). Dots represent the average values of every site. Source data are provided as a Source Data file.

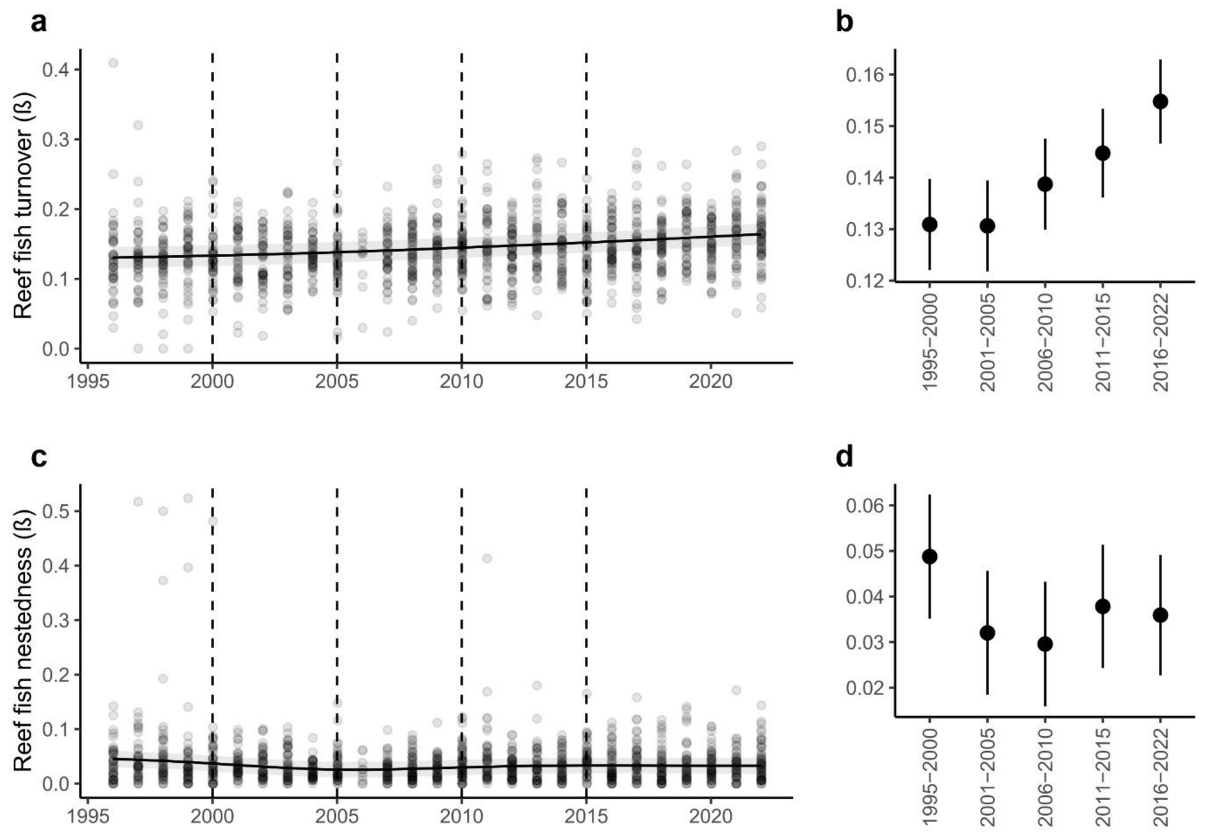

Supplementary Figure 6. **Reef fish year-to-year Sørensen dissimilarity (β diversity) in the Great Barrier Reef under presence and absence data.** Trends are the predicted values ± 95% CIs of the long-term changes in the reef fish Sørensen dissimilarity of 92 reefs (three sites per reef per year) from the hierarchical generalized additive mixed model (HGAM). Sørensen dissimilarity is separated into two components accounting for the dissimilarity derived solely from turnover (**a**) and (**b**) and the dissimilarity derived from nestedness (**c**) and (**d**). Transparent dots in (**a**) and (**c**) represent the mean values of sites. Predicted values of each time period from the generalized linear mixed model (GLMM) are shown in (**b**) and (**d**) for the GBR model where points represent the mean of the predicted values and lines represent the 95% CIs. Source data are provided as a Source Data file.

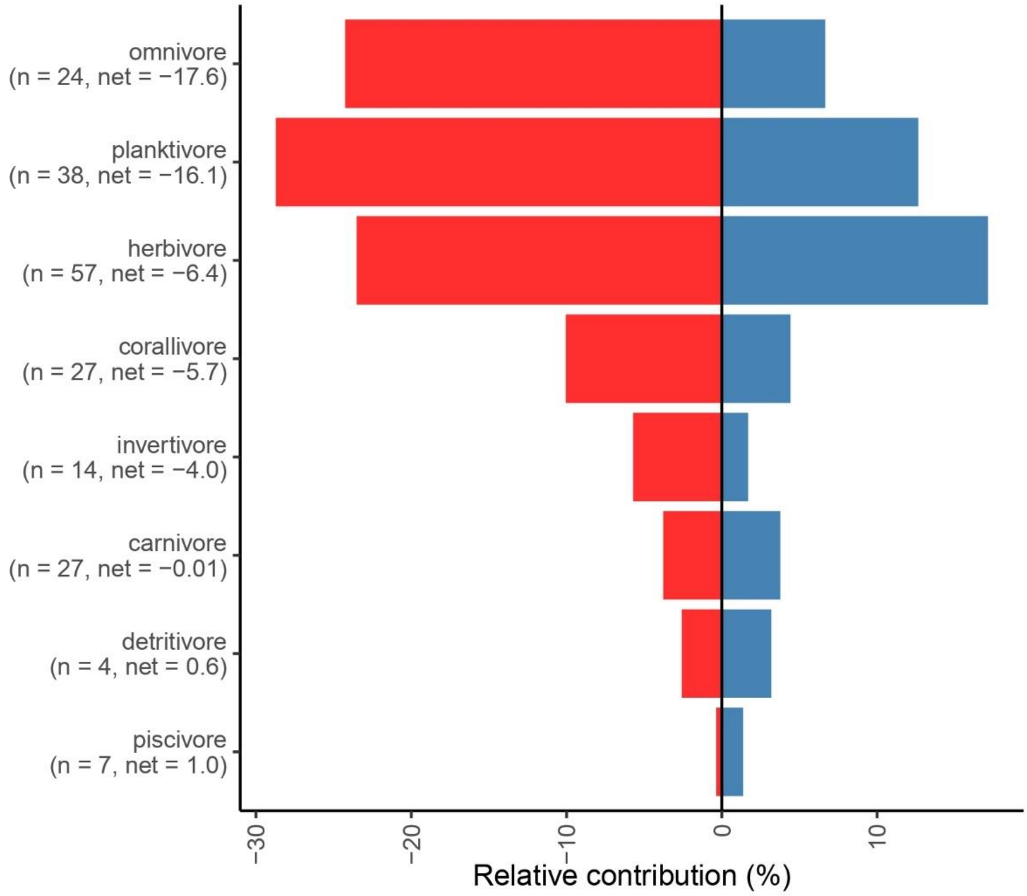

Supplementary Figure 7. **Contribution of reef fish functional groups to community dissimilarity between the initial and last period in the Great Barrier Reef (GBR; without reefs in the Capricorn-Bunker sector).** Vales are based on the similarity percentage (SIMPER) analysis in the Great Barrier Reef without Capricorn Bunker sector. Bars are species-level contributions summed by functional groups for species that increased (blue) or decreased (red) in abundance between the initial (1995-2000) and last (2016-2022) period. The initial period of Innisfail (IN) and Pompey (PO) is from 2006 to 2010. Trophic groups are ordered in descending order according to the greatest net negative contribution (%). Number of species (*n* =) and the net contribution to community dissimilarity (*net* =) for each functional group are shown in brackets. Source data are provided as a Source Data file.

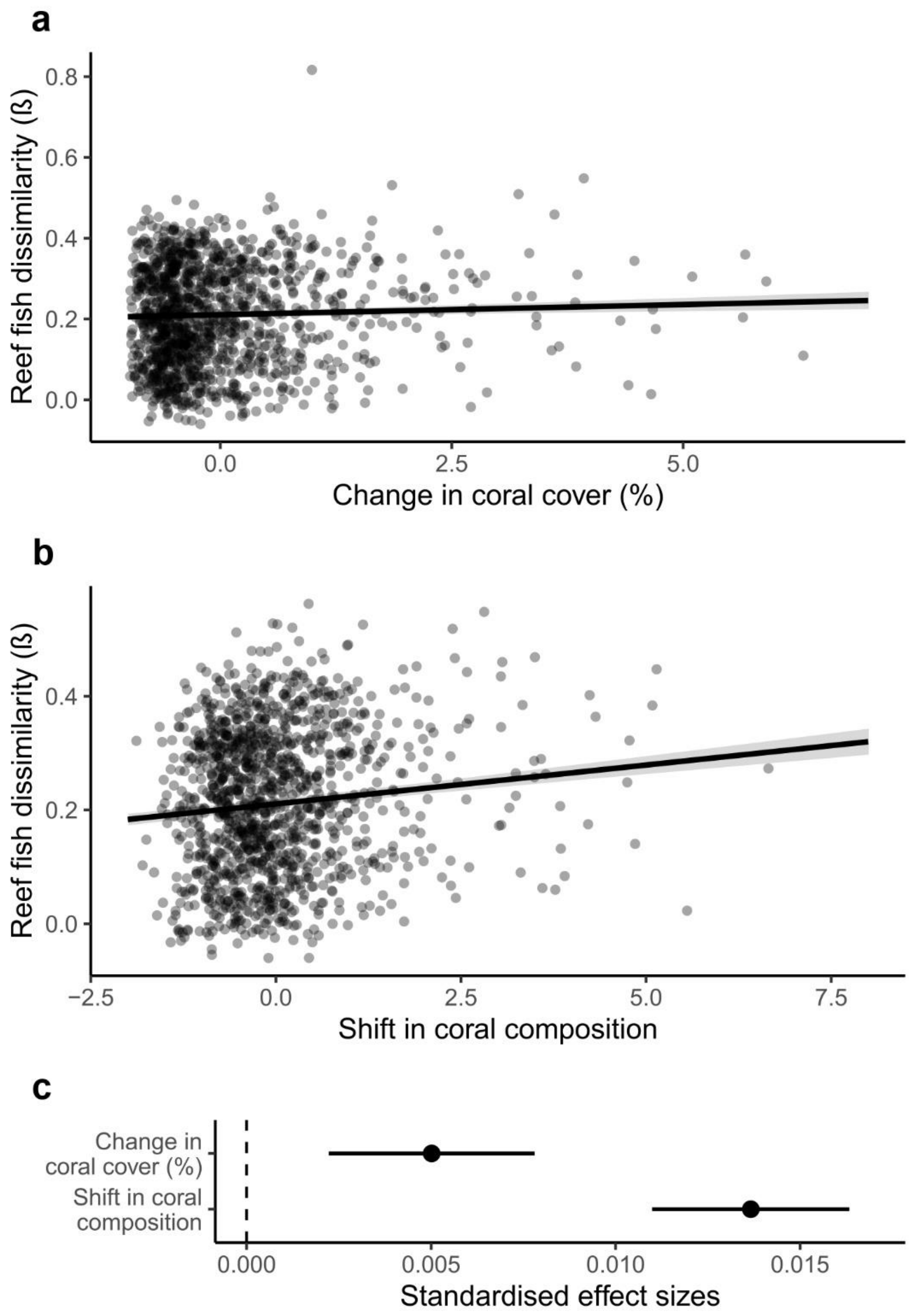

Supplementary Figure 8. **Reef fish dissimilarity (*β* diversity) correlates more strongly with shifts in coral composition than changes in coral cover (%).** Standardized predicted values of reef fish dissimilarity (*n* reefs = 92; three sites per reef per year) for (**a**) change in coral cover (%) and (**b**) shift in coral composition. Shadow bands in (**a**) and (**b**) represent the 95% CIs. Dots in (**a**) and (**b**) represent sites, and lines represent the generalised linear mixed models (GLMM) fitted by the reef fish dissimilarity. 'Reef' and 'shelf position' were used as random effects in our models. Points in (**c**) represent means of the effect sizes of the GLMM and lines the 95% CIs. Source data are provided as a Source Data file.

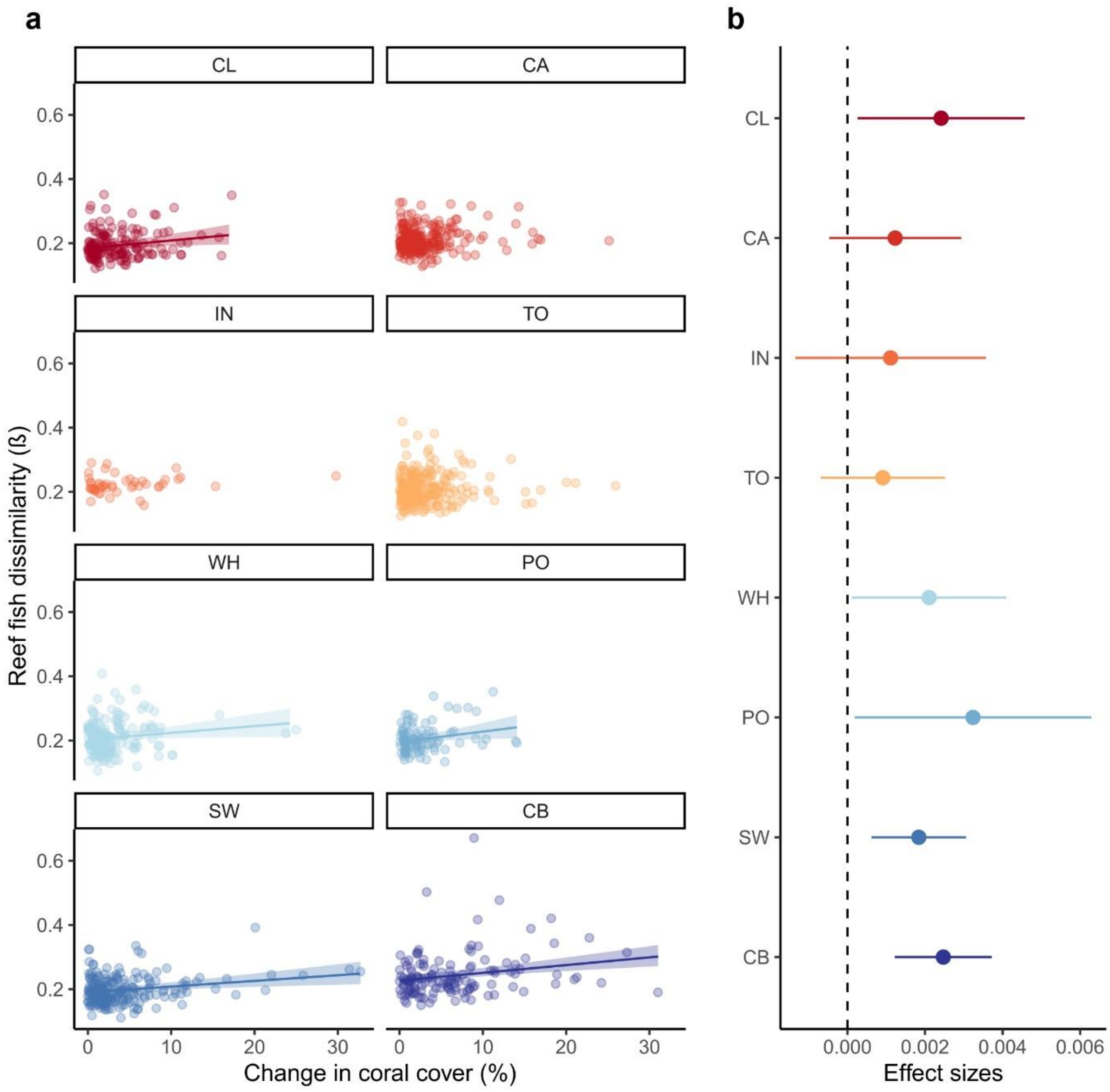

Supplementary Figure 9. **Reef fish dissimilarity (β diversity) and change in coral cover (%) across latitudinal sectors.** In (**a**) we show the predicted values of reef fish turnover (non-standardized) by the change in coral cover. 'Reef' and 'shelf position' were used as random effects in our models. Shadow bands in (**a**) represent the 95% CIs. Sample size of the 92 reefs (three sites for each reef per year) by latitudinal sector in (**a**): CL (*n* reefs = 8), CA (*n* reefs = 11), IN (*n* reefs = 7), TO (*n* reefs = 18), WH (*n* reefs = 9), PO (*n* reefs = 12), SW (*n* reefs = 17), CB (*n* reefs = 10). Dots in (**b**) represent the mean effect size (non-standardised) of shifts in coral composition on reef fish dissimilarity (β) for each latitudinal sector from the generalised linear mixed models (GLMM); lines represent 95% CIs. We assume a significant effect in (**b**) if the 95% CIs do not overlap zero (vertical dotted line). Codes are latitudinal sectors: Cooktown-Lizard Island (CL), Cairns (CA), Innisfail (IN), Townsville (TO), Whitsunday (WH), Pompey (PO), Swain (SW) and Capricorn-Bunker (CB) ordered in descending from lower to higher latitudes (from red to blue, respectively). Source data are provided as a Source Data file.

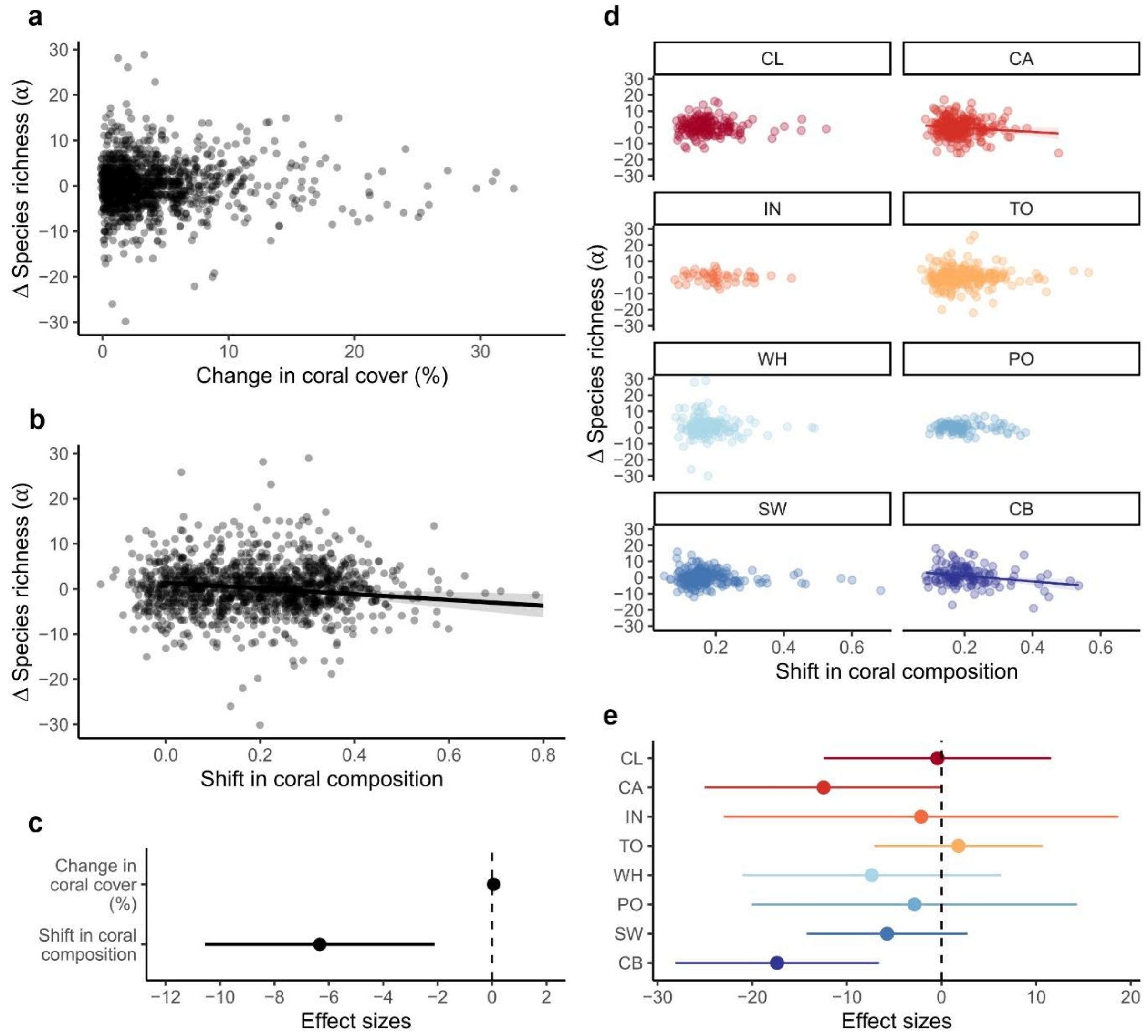

Supplementary Figure 10. **Change of reef fish species richness (α diversity) correlates with shifts in coral composition but not with changes in coral cover (%).** Predicted values of the Δ reef fish species richness (*α* diversity) for (**a**) change in coral cover (%) and (**b**) shift in coral composition (*n* reefs = 92 reefs; three sites per reef per year) . Dots in (**a**) and (**b**) represent sites and lines represent the mixed models fitted by the change of reef fish species richness. Dots in (**c**) represent means of the standardised effect sizes of the general linear mixed model (GLMM) and lines the 95% CIs. 'Reef' and 'shelf position' were used as random effects in our models. Dots in (**d**) represent the mean effect size (non-standardized) of shifts in coral composition on the change of reef fish species richness for each latitudinal sector from the GLMM; lines represent the 95% CIs. Sample size of the 92 reefs (three sites per reef per year) by latitudinal sector in (d): CL (*n* reefs = 8), CA (*n* reefs = 11), IN (*n* reefs = 7), TO (*n* reefs = 18), WH (*n* reefs = 9), PO (*n* reefs = 12), SW (*n* reefs = 17), CB (*n* reefs = 10). Shadow bands in (**a**), (**b**) and (**d**) represent the 95% CIs. In (**c**) and (**d**) we assume a significant effect if the 95% CIs do not overlap zero (vertical dotted line). Codes in (**d**) are latitudinal sectors: Cooktown-Lizard Island (CL), Cairns (CA), Innisfail (IN), Townsville (TO), Whitsunday (WH), Pompey (PO), Swain (SW) and Capricorn-Bunker (CB) ordered in descending from lower to higher latitudes. Source data are provided as a Source Data file.
