# Supplementary material for: Emergent patterns of reef fish diversity correlate with coral assemblage shifts along the Great Barrier Reef
Source: Nat Commun. 2025 Jan 13;16:303. doi: 10.1038/s41467-024-55128-7 (PMC11729903; doi:10.1038/s41467-024-55128-7)
Supplement: Supplementary file 3 — Reporting Summary [file 41467_2024_55128_MOESM3_ESM.pdf]

nature portfolio

Corresponding author(s): Francisco Javier Gonzalez Barrios

Last updated by author(s): Sep 23, 2024

Reporting Summary

Nature Portfolio wishes to improve the reproducibility of the work that we publish. This form provides structure for consistency and transparency in reporting. For further information on Nature Portfolio policies, see our Editorial Policies and the Editorial Policy Checklist.

Statistics

For all statistical analyses, confirm that the following items are present in the figure legend, table legend, main text, or Methods section.

n/a	Confirmed	
☐	☒	The exact sample size (*n*) for each experimental group/condition, given as a discrete number and unit of measurement
☐	☒	A statement on whether measurements were taken from distinct samples or whether the same sample was measured repeatedly
☐	☒	The statistical test(s) used AND whether they are one- or two-sided<br>*Only common tests should be described solely by name; describe more complex techniques in the Methods section.*
☐	☒	A description of all covariates tested
☐	☒	A description of any assumptions or corrections, such as tests of normality and adjustment for multiple comparisons
☐	☒	A full description of the statistical parameters including central tendency (e.g. means) or other basic estimates (e.g. regression coefficient) AND variation (e.g. standard deviation) or associated estimates of uncertainty (e.g. confidence intervals)
☒	☐	For null hypothesis testing, the test statistic (e.g. *F*, *t*, *r*) with confidence intervals, effect sizes, degrees of freedom and *P* value noted<br>*Give P values as exact values whenever suitable.*
☒	☐	For Bayesian analysis, information on the choice of priors and Markov chain Monte Carlo settings
☐	☒	For hierarchical and complex designs, identification of the appropriate level for tests and full reporting of outcomes
☐	☒	Estimates of effect sizes (e.g. Cohen's *d*, Pearson's *r*), indicating how they were calculated

Our web collection on statistics for biologists contains articles on many of the points above.

Software and code

Policy information about availability of computer code

Data collection	No software was used for data collection
Data analysis	β metrics were estimated using beta.pair.abund function in the betapart package (Baselga & Orme, 2012). The permutational multivariate analysis was performed using the adonis function from the vegan package as well as the SIMPER and nMDS multivariate analyses (Oksanen et al. 2020). HGAMs were generated using the gam function from the mgcv package (Wood 2017) and GLMMs were generated with the lmer function from the lme4 package (Bates et al. 2015). All models, analyses and figures were generated in R version 4.3.0 (R Core Team, 2023).

For manuscripts utilizing custom algorithms or software that are central to the research but not yet described in published literature, software must be made available to editors and reviewers. We strongly encourage code deposition in a community repository (e.g. GitHub). See the Nature Portfolio guidelines for submitting code & software for further information.

Data

Policy information about availability of data

All manuscripts must include a data availability statement. This statement should provide the following information, where applicable:

- Accession codes, unique identifiers, or web links for publicly available datasets
- A description of any restrictions on data availability
- For clinical datasets or third party data, please ensure that the statement adheres to our policy

All data used in this manuscript are publicly available upon request from the Australian government's Australian Institute of Marine Science: monitoring@aims.gov.au; We provide R code associated with this study at an open source repository (https://github.com/JavierGonzalezBarrios/Diversity-patterns-

changes)

Research involving human participants, their data, or biological material

Policy information about studies with human participants or human data. See also policy information about sex, gender (identity/presentation), and sexual orientation and race, ethnicity and racism.

Reporting on sex and gender	*Use the terms sex (biological attribute) and gender (shaped by social and cultural circumstances) carefully in order to avoid confusing both terms. Indicate if findings apply to only one sex or gender; describe whether sex and gender were considered in study design; whether sex and/or gender was determined based on self-reporting or assigned and methods used. Provide in the source data disaggregated sex and gender data, where this information has been collected, and if consent has been obtained for sharing of individual-level data; provide overall numbers in this Reporting Summary. Please state if this information has not been collected. Report sex- and gender-based analyses where performed, justify reasons for lack of sex- and gender-based analysis.*
Reporting on race, ethnicity, or other socially relevant groupings	*Please specify the socially constructed or socially relevant categorization variable(s) used in your manuscript and explain why they were used. Please note that such variables should not be used as proxies for other socially constructed/relevant variables (for example, race or ethnicity should not be used as a proxy for socioeconomic status). Provide clear definitions of the relevant terms used, how they were provided (by the participants/respondents, the researchers, or third parties), and the method(s) used to classify people into the different categories (e.g. self-report, census or administrative data, social media data, etc.) Please provide details about how you controlled for confounding variables in your analyses.*
Population characteristics	*Describe the covariate-relevant population characteristics of the human research participants (e.g. age, genotypic information, past and current diagnosis and treatment categories). If you filled out the behavioural & social sciences study design questions and have nothing to add here, write "See above."*
Recruitment	*Describe how participants were recruited. Outline any potential self-selection bias or other biases that may be present and how these are likely to impact results.*
Ethics oversight	*Identify the organization(s) that approved the study protocol.*

Note that full information on the approval of the study protocol must also be provided in the manuscript.

Field-specific reporting

Please select the one below that is the best fit for your research. If you are not sure, read the appropriate sections before making your selection.

☐ Life sciences ☐ Behavioural & social sciences ☒ Ecological, evolutionary & environmental sciences

For a reference copy of the document with all sections, see nature.com/documents/nr-reporting-summary-flat.pdf

Ecological, evolutionary & environmental sciences study design

All studies must disclose on these points even when the disclosure is negative.

Study description	We analysed how and why patterns of reef fish diversity have changed by examining local diversity (α diversity) and species turnover (β diversity) from 1995 to 2022 along a large latitudinal gradient (>1200 km) of the Great Barrier Reef (GBR). We examine whether fluctuations in coral cover or the composition of coral assemblages correlate with patterns in reef fish diversity across large spatial extents. Our work provides fundamental insight into the extent to which classic macroecological patterns are changing in the Anthropocene.
Research sample	The reef benthos, and associated fish communities, were surveyed using underwater visual census techniques. Surveys were structured hierarchically. At each of the 98 sampled reefs, 3 sites were surveyed. Sites are at least 250 m apart where possible. Within each site, five permanent transect 5 x 50 m wide for fish and 1 x 50 m wide for benthos, laid along the reef slope parallel to the reef crest at about 6-9 m depth.
Sampling strategy	AIMS reef monitoring programs use Standard Operational Procedures to ensure consistent methodology through time. This ensures that the data we are reporting on has been collected in a consistent, rigorous way, enabling robust appraisal of any changes in reef condition indicators through time. The methods used are globally accepted by the scientific community.
Data collection	At each site, five permanent transects 5 m x 50 m wide were used to survey the abundance of large diurnal non-cryptobenthic reef-associated fishes from nine families covering a total of 198 reef fish species. Damselfishes (47 species) were counted separately using 1 m x 50 m wide belts along the same transects. Over the same transect, photo transects were generated to catalogue benthic assemblages, by taking an image every 1 m along the 50 m transects (50 images per transect), from which forty were randomly selected. The percentage cover of benthic groups was estimated from images using five points per image (n = 200 points per transect) and organisms were identified to the finest taxonomic resolution possible (usually genera) and placed into morphological groups.
Timing and spatial scale	The LTMP has monitored 92 reefs over a 28-year period (1995 to 2022), across eight latitudinal sectors (Cooktown-Lizard Island, Cairns, Innisfail, Townsville, Whitsunday, Pompey, Swain and Capricorn Bunker) spanning >1100 km (from 14°S to 24°S) of the GBR

	Between 1995 and 2005, 45 reefs were monitored annually. Then, from 2006 to 2020, 82 reefs were surveyed biennially and 10 annually. Six of the 8 sectors have included survey reefs throughout the whole time series, while 2 sectors (Innisfail, Pompey) were added in 2006.
Data exclusions	In 2019 the LTMP included an additional ten reefs added, extending surveys to the Far Northern GBR which to that point had not been surveyed. We did not included these reef sites into our analyses due to the short temporal span of sampling.
Reproducibility	A full description of the methodologies used is provided in the Methods. All data used in this manuscript are publicly available upon request from the Australian government's Australian Institute of Marine Science: monitoring@aims.gov.au
Randomization	Fixed site surveys, in which reef fish and benthos on the reef surface are surveyed at three sites, in a habitat that is standardised across reefs. Underwater visual survey counts were started from the beginning to the end for each transect. Ensuring each site is permanently marked helps reduce variation in the data due to sampling, and enables us to detect changes through time, which is the primary goal of monitoring. Fixed site surveys are designed to detect reef community changes in more detail and at finer taxonomic resolution than what can be provided by random surveyed points.
Blinding	Blinding was not necessary as these were surveys of wild communities of benthic organisms and fish.

Did the study involve field work? ☒ Yes ☐ No

Field work, collection and transport

Field conditions	Field conditions in the sea were appropriate and allowed to conduct monitoring surveys.
Location	The study was conducted at reef sites in a standardized reef slope habitat between 6 to 9 m depth throughtur the Australia' Great Barrier Reef (from 14°S to 24°S and 145°E to 153°E).
Access & import/export	All surveys were conducted with research permit clearance and collaborative with the Great Barrier Reef Marine Park Authority.
Disturbance	All surveys were observational only.

Reporting for specific materials, systems and methods

We require information from authors about some types of materials, experimental systems and methods used in many studies. Here, indicate whether each material, system or method listed is relevant to your study. If you are not sure if a list item applies to your research, read the appropriate section before selecting a response.

Materials & experimental systems

n/a	Involved in the study
☒	☐ Antibodies
☒	☐ Eukaryotic cell lines
☒	☐ Palaeontology and archaeology
☐	☒ Animals and other organisms
☒	☐ Clinical data
☒	☐ Dual use research of concern
☒	☐ Plants

Methods

n/a	Involved in the study
☒	☐ ChIP-seq
☒	☐ Flow cytometry
☒	☐ MRI-based neuroimaging

Animals and other research organisms

Policy information about studies involving animals; ARRIVE guidelines recommended for reporting animal research, and Sex and Gender in Research

Laboratory animals	The study did not involve laboratory animals.
Wild animals	All wild animal work was observational (visual surveys).
Reporting on sex	Sex was not considered as past of this study.
Field-collected samples	The study did not involve samples collected from the field.
Ethics oversight	No ethical clearance was necessary as the study was observational.

Note that full information on the approval of the study protocol must also be provided in the manuscript.

Plants

Seed stocks	*Report on the source of all seed stocks or other plant material used. If applicable, state the seed stock centre and catalogue number. If plant specimens were collected from the field, describe the collection location, date and sampling procedures.*
Novel plant genotypes	*Describe the methods by which all novel plant genotypes were produced. This includes those generated by transgenic approaches, gene editing, chemical/radiation-based mutagenesis and hybridization. For transgenic lines, describe the transformation method, the number of independent lines analyzed and the generation upon which experiments were performed. For gene-edited lines, describe the editor used, the endogenous sequence targeted for editing, the targeting guide RNA sequence (if applicable) and how the editor was applied.*
Authentication	*Describe any authentication procedures for each seed stock used or novel genotype generated. Describe any experiments used to assess the effect of a mutation and, where applicable, how potential secondary effects (e.g. second site T-DNA insertions, mosiacism, off-target gene editing) were examined.*
